# Determination and Pharmacokinetic Study of Three Diterpenes in Rat Plasma by UHPLC-ESI-MS/MS after Oral Administration of *Rosmarinus officinalis* L. Extract

**DOI:** 10.3390/molecules22060934

**Published:** 2017-06-04

**Authors:** Liqian Wang, Chunli Gan, Zhibin Wang, Lu Liu, Mingjie Gao, Qian Li, Chunjuan Yang

**Affiliations:** 1 Department of Pharmaceutical Analysis and Analytical Chemistry, College of Pharmacy, Harbin Medical University, No. 157 Baojian Road, Nangang District, Harbin 150081, Heilongjiang, China; wangliqian93@163.com (L.W.); a1534064875@163.com (L.L.); gaomingjie8888@163.com (M.G.), liqian@ems.hrbmu.edu.cn (Q.L.); 2 Department of Medicinal Chemistry and Natural Medicine Chemistry, College of Pharmacy, Harbin Medical University, No. 157 Baojian Road, Nangang District, Harbin 150081, Heilongjiang, China; chunligan@126.com; 3 Key Laboratory of Chinese Materia Medica (Ministry of Education), Heilongjiang University of Chinese Medicine, Harbin 150040, Heilongjiang, China; wzbmailbox@126.com

**Keywords:** *Rosmarinus officinalis* L., UHPLC/MS/MS, diterpenes, pharmacokinetics

## Abstract

*Rosmarinus officinalis* L. is commonly used as a spice and flavoring agent. Diterpenes are the main active compounds of *R. officinalis*. An Ultra High Performance Liquid Chromatography-Tandem Mass Spectrometry (UHPLC-ESI-MS/MS) method was developed for the determination of carnosol, rosmanol, and carnosic acid isolated from *R. officinalis* in rat plasma, and applied to a pharmacokinetic study after oral administration of *R. officinalis* extract. Sample preparation involved a liquid-liquid extraction of the analytes with ethyl acetate. Butylparaben was employed as an internal standard (I.S.). Chromatographic separation was carried out on a C_18_ column (ACQUITY UPLC^®^ HSS T3, 1.8 μm, 2.1 mm × 100 mm) with a gradient system consisting of the mobile phase solution A (0.1% formic acid in water) and solution B (acetonitrile) at the flow rate of 0.3 mL/min. The quantification was obtained using multiple reaction monitoring (MRM) mode with electrospray ionization (ESI). The UHPLC-MS/MS assay was validated for linearity, accuracy, precision, extraction recovery, matrix effect and stability. This study described a simple, sensitive and validated UHPLC-MS/MS method for the simultaneous determination of three diterpene compounds in rat plasma after oral administration of *R. officinalis* extract, and investigated on their pharmacokinetic studies as well.

## 1. Introduction

*Rosmarinus officinalis* L., known as rosemary, belongs to the *Lamiaceae* family, which is native to the Mediterranean region [1]. Nowadays, it is an abundant herb commonly used as a spice and flavoring agent that grows wildly in many diverse areas of southern China. Many studies have shown that rosemary extract has antibacterial, antioxidant [2,3,4,5,6], antidiabetic [7], antitumor [8], anti-inflammatory and antinociceptive activities [9]. Constituents like carnosol, rosmanol and carnosic acid are the main active ingredients of this plant [10,11,12].

**Figure 1:**
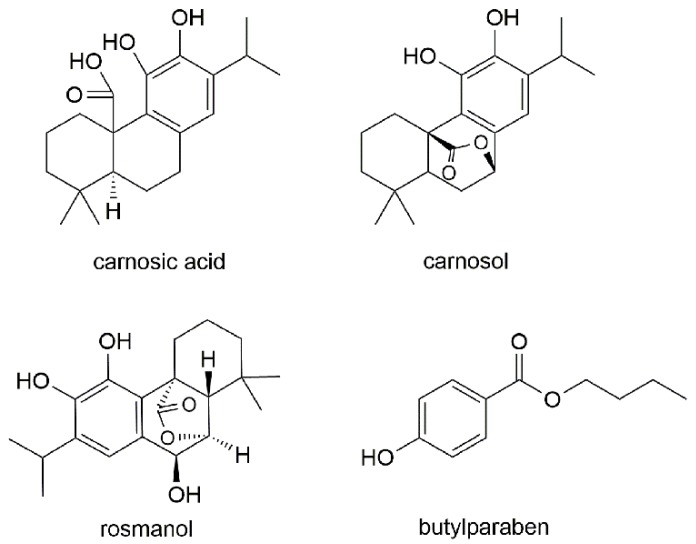
The chemical structures of the three analytes and I.S.

In our previous investigation, three diterpene compounds (rosmanol, carnosol and carnosic acid) were isolated from the leaves of rosemary. Carnosic acid (Figure 1) is a tricyclic diterpene possessing a dihydroxylated polyphenolic ring and a free carboxylic acid group in its molecular structure, which may be the structural basis for its high antioxidant activity [11]. In addition, carnosic acid is reported to exhibit antitumor effects in vitro and in vivo [13]. Carnosol (Figure 1) is a derivative of carnosic acid also possessing various kinds of pharmacological activities such as antioxidant, anti-inflammatory, and antitumor activities [14,15]. Carnosol has been proposed as an antitumor agent in several studies [16]. Rosmanol (Figure 1), is a phenolic diterpene reported to have high antioxidant, anti-inflammatory and anti-tumorogenic activities. Rosmanol was also found to be a more potent cytotoxic agent compared to carnosol and rosmarinic acid according to Cheng’s study [17].

Various analytical methods, including high performance liquid chromatography (HPLC) and UHPLC-MS/MS have been used in the qualitative or quantitative analysis of rosemary in vitro. To the best of our knowledge, there are two published articles on the pharmacokinetic study of carnosic acid. Doolaege et al. developed a LC-MS method to evaluate the bioavailability of carnosic acid in rats using single reaction monitoring (SRM) [18] and Yan et al. established a HPLC method to determine carnosic acid in rat plasma [19]. However, this work represents the first time a sensitive and selective UHPLC-ESI-MS/MS method has been developed and validated for the simultaneous determination of carnosol, rosmanol, and carnosic acid in rat plasma after oral administration of the rosemary extract was established. The method was applied to a pharmacokinetic study after the oral administration of rosemary extract to rats. We expect that the results of this study will provide some useful references for the further pharmacological study of rosemary.

## 2. Results and Discussion

### 2.1. Mass Spectra

**Figure 2:**
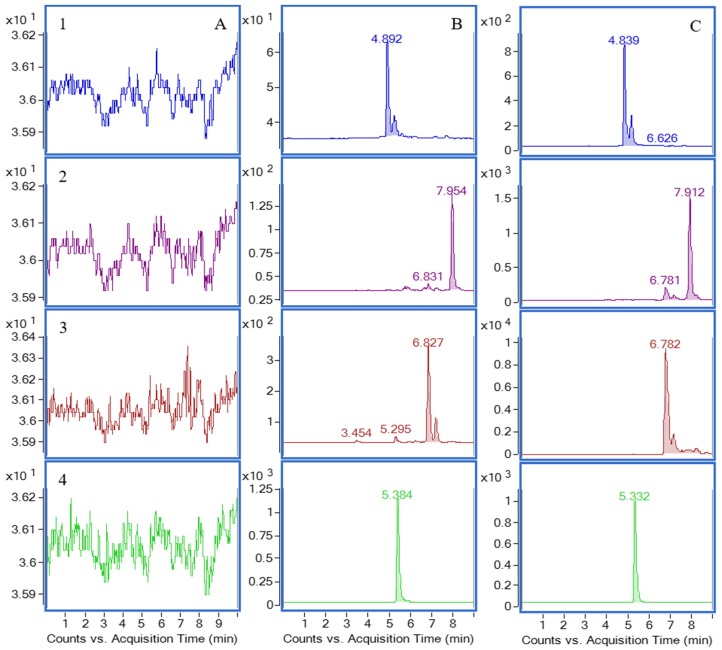
Chromatograms of the three components and I.S. in rat plasma: (**A**) blank plasma; (**B**) blank plasma spiked with the three analytes (MQC) and I.S.; (**C**) plasma sample obtained at 0.5 h from a rat after oral administration of rosemary extract, channel 1 for rosmanol (568.3 ng/mL); channel 2 for carnosic acid (27,948 ng/mL); channel 3 for carnosol (1825.1 ng/mL); channel 4 for I.S.

The first step of the method was to select precursor ions and product ions of the analytes and the I.S. for the MRM mode analysis. Other parameters were also optimized for achieving a good sensitivity. In our experiment, it was discovered that the response of diterpenes in negative ion mode was higher than that in positive ion mode, thus negative ion mode was employed. Figure 2 shows the product ion scan spectra of the analytes and the I.S. The precursor ion of carnosic acid was at *m/z* 331.2 Da ([M − H]^−^), and the product ion peak at *m/z* 287.2 Da was attributable to the typical CO_2_ loss (44 Da). The loss of CH(CH_3_)_2_ (43 Da) from the product ion (287.2 Da) yielded a fragment ion at *m/z* 244.1 Da. The precursor ion of carnosol was at *m/z* 329.1 Da ([M − H]^−^), and the product ion peak at *m/z* 285.1 was also attributable to CO_2_ loss (44 Da).

The precursor ion of rosmanol was at m/z 345.1 Da ([M − H]^−^), and the product ion peak at *m/z* 283.0 was attributable to one molecule of CO_2_ and one molecule of H_2_O loss (62 Da). The precursor ion of butylparaben (C_11_H_14_O_3_) was at *m/z* 193.0 Da ([M − H]^−^), and the product ion peak at *m/z* 92.0 was attributable to a COO(CH_2_)_3_CH_3_ loss (101 Da). Table 1 shows the MS/MS transitions and energy parameters of all the compounds.

### 2.2. Chromatography

The chromatographic conditions were optimized to improve the peak shape, increase the signal response of the analytes and shorten the run time. The mobile phase systems of acetonitrile-water and methanol-water in various proportions were tested. The response of analytes using acetonitrile-water was obviously higher than that with methanol-water. It is well known that the ionization in ESI mode occurs in the solution state. The additives may have a significant influence on the response of the analytes. Different buffers including formic acid (0.1%), ammonium acetate (2 and 5 mm) and acetic acid (0.2%) were chosen to optimize the mobile phase to produce the best response, sensitivity and separation efficiency. As a result, the acetonitrile-water (0.1% formic acid) system with optimized gradient elution showed higher elution strength, a good separation and abundant signal response in negative ion scan mode. The high chromatographic resolution of the UHPLC system was enabled to increase peak capacity. Meanwhile the mobile phase composition, flow rate and column temperature had been adjusted to obtain an acceptable resolution observed. Best chromatographic separations were achieved at 30 °C with a gradient mobile phase consisting of acetonitrile-water (0.1% formic acid) at a flow rate of 0.3 mL/min with an acceptable run time of 9 min. While the both run times in the two published articles were 30 min [18,19]. Butylparaben was finally selected as the I.S. because of its suitable retention time and clear resolution in our mobile phase.

### 2.3. Selection of Extraction Method

Liquid-liquid extraction (LLE), protein precipitation (PPT) and solid phase extraction (SPE) were investigated in our study. SPE with Waters Oasis HLB cartridges (Milford, MA, USA) was expensive, complicated and time-costing, not suitable for multi-sample analysis. Liquid-liquid extraction was chosen as the method for sample preparation because this technique could produce not only purified but also concentrated samples. Several extraction solvents such as ethyl acetate, chloroform and isopropyl alcohol-hexane (9:1, *v/v*) were investigated. Because of its high extraction efficiency, low noise level and better repeatability, ethyl acetate was selected as the extraction solvent.

### 2.4. Method Validation

#### 2.4.1. Selectivity

The selectivity of the method towards endogenous plasma matrix was evaluated with plasma from six rats. Four channels were used for recording and the retention times of carnosol, rosmanol, carnosic acid and I.S. were 6.78, 4.84, 7.91, and 5.33 min, respectively. The typical chromatograms of the blank plasmas, plasma samples spiked with the lower limits of quantification (LLOQ) analytes and the I.S., and the 0.5 h plasma sample from a rat after oral administration of *R. officinalis* extract are shown in Figure 2. All the peaks of the analytes and the I.S. were detected with excellent resolution as well as peak shapes. The analytes could be easily differentiated from the rat plasma matrix and quantitatively determined at the LLOQ level.

#### 2.4.2. Linearity and Lower Limit of Quantification

The typical equation of calibration curves and linearity ranges for the three analytes are shown in Table 2. All of the correlation coefficients were higher than 0.99. The results showed that there was excellent correlation between the ratio of peak area and concentration for each compound within the linearity ranges. The results for LLOQs are also shown in Table 2. The linear calibration ranges were 10.75~32,250 ng/mL for carnosic acid, 1.453~4360 ng/mL for carnosol, 1.700~5100 ng/mL for rosmanol. Compared with the two published articles [18,19], the LLOQ (10.75 ng/mL) is lower than that of the previous analytical methods (265 μg/mL and 500 μg/mL).

#### 2.4.3. Accuracy and Precision

In this assay, the intra-day and inter-day precisions (RSD%) and accuracys (RE%) were evaluated by determination of QC samples at three concentration levels (LQC, MQC, HQC) of the three analytes on the same day and on three consecutive days, respectively. As described in Table 3, the intra-day and inter-day precisions were less than 20%. Accuracy values ranged from −6.06 to 8.88%. These results suggested that the developed method was precise, accurate and reproducible.

#### 2.4.4. Stability

The stability of the three diterpenes was assessed under various conditions (Table 4). The variations in all stability studies were within ±15%, indicating that the three analytes were all stable in rat plasma after three freeze-thaw cycles, at room temperature for 4 h. Post-preparative stability of the analytes also showed no significant degradation when the extracted samples were kept at 4 °C for 12 h. Moreover, all the investigated compounds were stable for 2 weeks when kept frozen at −20 °C.

#### 2.4.5. Matrix Effect and Recovery

The average extraction recoveries and matrix effects of the QC samples are summarized in Table 5. Recoveries of the three diterpenes at all levels were higher than 84.10% and that of the I.S. was about 82.64%, which was adequate and acceptable for the PK studies.

Meanwhile, the matrix effects of I.S. and QC samples of the three diterpene compounds at three concentrations were observed to range from 97.34 to 105.3%, respectively. The data above suggested that ion suppression or enhancement from plasma matrix is negligible for this method.

### 2.5. Application to a Pharmacokinetic Study

**Figure 3:**
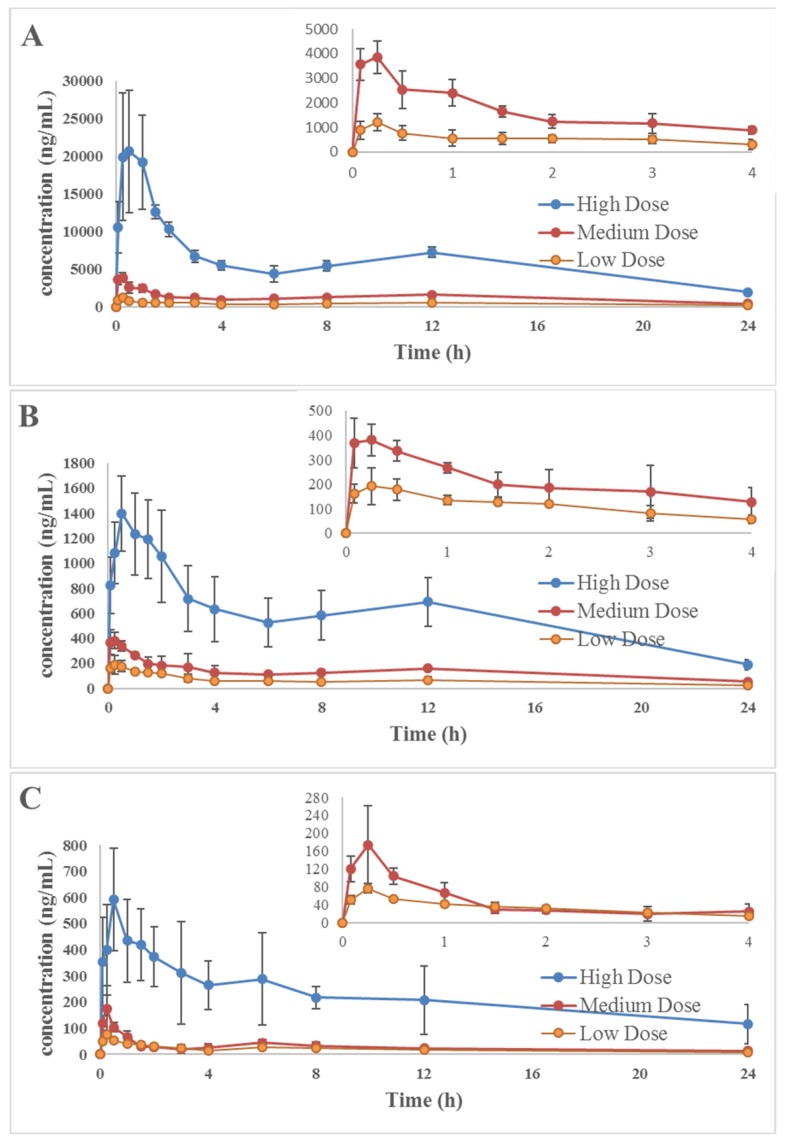
Mean concentration-time profiles of carnosic acid (**A**); carnosol (**B**); rosmanol (**C**) in rat plasma after oral administration of rosemary. Each point represents the mean ± SD (*n* = 6).

The validated method was successfully applied to pharmacokinetic studies of three diterpenes components after oral administration of rosemary at three different doses (0.25, 0.82, 2.45 g/kg). *R. officinalis* is commonly used as a spice and flavoring agent. So the amount of rosemary used in daily is always varied according to people’s tastes. Therefore, we set low, medium and high doses in this study. The mean plasma concentration-time curves (*n* = 6) of the analytes are shown in Figure 3.

The main pharmacokinetic parameters of the analytes were calculated with DAS 2.0 (Shanghai, China) by non-compartmental analysis. The pharmacokinetic parameters including half-time (*t*_1/2_), the maximum plasma concentration (*C*_max_), the time to reach the maximum concentrations (*t*_max_), elimination rate constants (*Ke*), area under concentration-time curve (AUC_0→t_) calculated by non-compartment model are presented in Table 6.

As seen from Table 6, the three analytes were rapidly absorbed, and they achieved *C*_max_ values between 0.2 and 1.00 h after oral administration, and then they were slowly eliminated as the plasma concentration was still much higher than LOQ after 12 h. The *C*_max_ values of low, medium, high dose groups were 1212 ± 344, 3955 ± 515, 27,504 ± 1881 ng/mL for carnosic acid, 216.6 ± 53.1, 413.3 ± 42.6, 1480 ± 266 ng/mL for carnosol, 77.20 ± 10.54, 187.9 ± 75.9 633.4 ± 118.5 ng/mL for rosmanol, respectively. And the *t*_max_ values of low, medium, high dose groups were 0.30 ± 0.11, 0.30 ± 0.11, 0.50 ± 0.31 h for carnosic acid, 0.40 ± 0.13, 0.27 ± 0.15, 0.70 ± 0.45 h for carnosol, 0.25 ± 0.00, 0.20 ± 0.18, 0.55 ± 0.27 h for rosmanol, respectively. As we can see from the data results, *C*_max_ and *t*_max_ values are increased accordingly with the increase in dosage. According to our research findings, the *t*_max_ value of carnosic acid was 0.3–0.5 h instead of 130 min once published. Different from other study, laboratory animals in our research were given *R. officinalis* extract not monomeric compound (purity ≥ 98%) [19]. Different drug types and complex components in Chinese traditional medicine may be the cause of the differernce of carnosic acid *t*_max_ value. As seen from Figure 3, the plasma concentration-time profiles of the different doses of carnosic acid, carnosol and rosmanol were similar. In the elimination phase, a double-peak phenomenon appeared on the mean plasma concentration-time profiles. The probably reason may be caused by redistribution and enterohepatic circulation after the analytes were excreted into the gastrointestinal tract through the bile.

## 3. Materials and Methods

### 3.1. Materials and Reagents

Reference standards of carnosol and rosmanol (Purity 98%, HPLC) were isolated from *R. officinalis* and their structures were confirmed by MS and HPLC. A reference standard of carnosic acid (purity 98%) were purchased from Chengdu Must Bio-technology co. (Chengdu, China). Butylparaben was purchased from Guangfu Fine Chemical Research Institute (Tianjin, China) and used as the internal standard (I.S.). The chemical structures of the three analytes and I.S. are shown in Figure 1. HPLC-grade methanol and acetonitrile were purchased from J&K MEDICAL (Beijing, China). Formic acid (HPLC-grade) was bought from DikmaPure (DIKMA, Lake ForestCA, USA). Ammonium acetate (HPLC-grade) was purchased from Kermel (Tianjin, China). All other reagents were of analytical grade. Ultrapure water was produced using a Milli-Q water purification system (Millipore, Molsheim, France).

### 3.2. Instruments and Analytical Conditions

**Figure 4:**
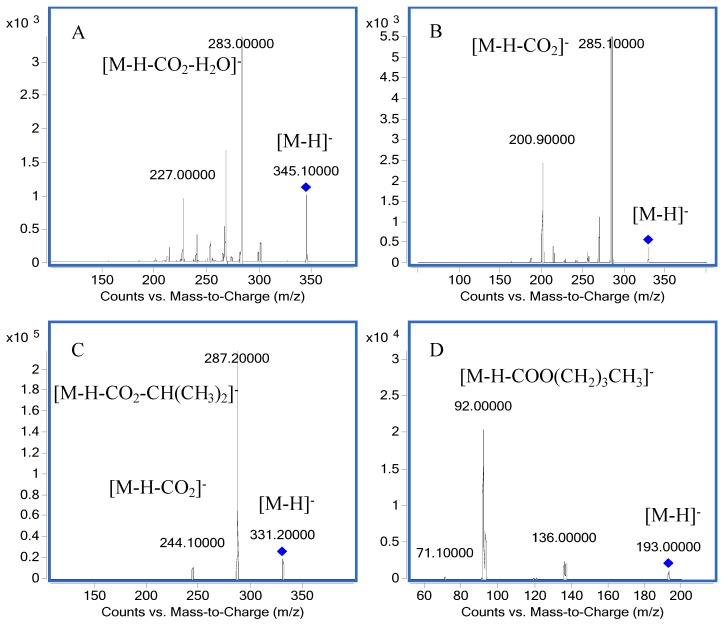
Product ion mass spectra of three analytes and I.S.: (**A**) rosmanol; (**B**) carnosol; (**C**) carnosic acid; (**D**) butylparaben.

**Figure 5:**
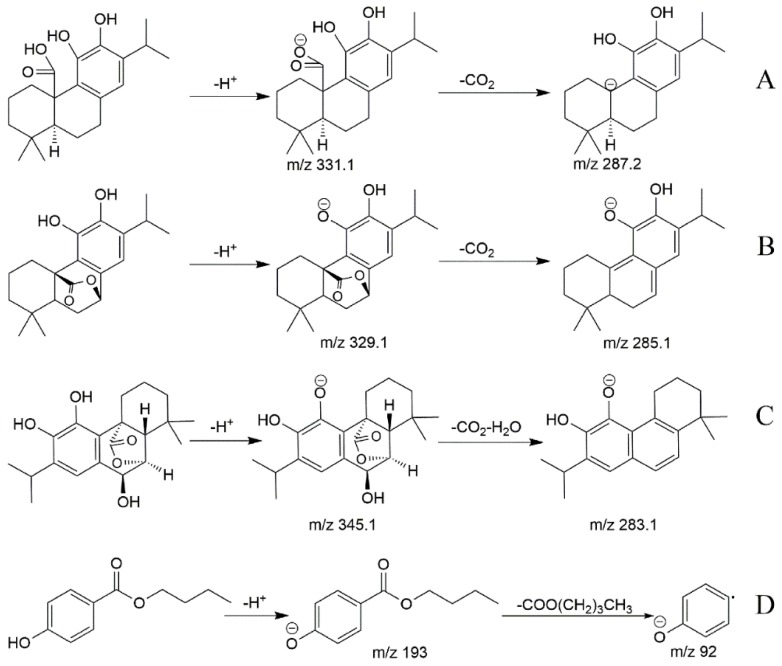
The possible fragmentation patterns of three analytes and I.S. in mass spectrometry: (**A**) carnosic acid; (**B**) carnosol; (**C**) rosmanol; (**D**) butylparaben.

The UHPLC-MS system (1290 series, Agilent Technologies, Santa Clara, CA, USA) consisted of an automatic degasser, an auto-sampler, and a quaternary pump, and was equipped with an electrospray ionization (ESI) interface. The chromatographic separation was carried out on a C_18_ column (Agilent ACQUITY UPLC^®^ HSS T3, 1.8 μm, 2.1 mm × 100 mm) with a gradient system consisting of mobile phase solution A (0.1% formic acid in water) and solution B (acetonitrile) at a flow rate of 0.3 mL/min. The gradient elution was programmed as follows: 0.0–6.0 min, 60%A–15%A; 6.0–8.0 min, 15%A–5%A; 8.0–9.0 min, 5%A–60%A. The column temperature was set at 30 °C. Each injection operated for 9 min and the injection volume was 10 μL. The detection was performed on a 6430 triple-quadrupole mass spectrometer (Agilent). The ESI source, negative ionization mode and multiple reaction monitoring (MRM) technique were employed. An Agilent Mass Hunter workstation was used to control the equipment and for the data acquisition and analysis. The quantification was obtained with precursor-product ion transitions at *m/z* 331.2→287.2 for carnosic acid, *m/z* 329.1→285.1 for carnosol, *m/z* 345.1→283.0 for rosmanol, and *m/z* 193.0→92.0 for butylparaben (I.S.), respectively. Qualifier ion transitions were at *m/z* 244.1 for carnosic acid, *m/z* 200.9 for carnosol, *m/z* 227.0 for rosmanol, and *m/z* 136.0 for butylparaben (I.S.), respectively. The fragment and collision energy are listed in Table 1. Product ion mass spectra of the three analytes and butylparaben (I.S.) are exhibited in Figure 4. The possible fragmentation patterns of the compounds are listed in Figure 5.

High purity (99.999%) nitrogen (N_2_) was used as the nebulizing gas and nitrogen (N_2_) was used as drying gas at a flow rate of 12 L/min. The mass spectrometer was operated at a capillary voltage of 4000 V, a desolvation temperature of 350 °C and a nebulizer pressure of 15 psi were employed.

### 3.3. Preparation of R. officinalis Extract in the Administration Solution

For the preparation of the extract, 100 g of dried leaves of *R. officinalis* were extracted under reflux with 1 L ethanol-water (80:20, *v/v*) for three times, 1 h for each time, and then filtrated. The combined filtrate was evaporated to dryness, and the residue was dissolved in water to get a concentration equivalent to 0.1 g/mL of the *R. officinalis* extract.

### 3.4. Preparation of Calibration Standards and Quality Control Samples

A mixed stock solution containing 645.0 μg/mL of carnosic acid, 43.60 μg/mL of carnosol and 51.00 μg/mL of rosmanol was prepared in methanol. A series of working standard solutions were obtained by successive dilution of the mixed stock solution with methanol. Similarly, the I.S. stock standard solution was diluted to a 2120 ng/mL working solution. Calibration standards were prepared by spiking 100 μL of the standard working solutions into 100 μL blank plasma to yield calibration concentrations of 10.75, 21.50, 53.75, 268.8, 1075, 5375, 16,125, 32,250 ng/mL for carnosic acid, 1.453, 2.907, 7.267, 36.33, 145.3, 726.7, 2180, 4360 ng/mL for carnosol and 1.700, 3.400, 8.500, 42.50, 170.0, 850.0, 2550, 5100 ng/mL for rosmanol, respectively. All the stock and working solutions were stored at −20 °C, and brought to room temperature before use. The QC samples were prepared in blank plasma at four different concentration levels, high QC (25,800/3488/4080 ng/mL), medium QC (1075/145.3/170.0 ng/mL), low QC (21.50/2.910/3.400 ng/mL), and lower limits of quantification (LLOQ) (10.75/1.453/1.700 ng/mL), for carnosic acid, carnosol and rosmanol, respectively.

### 3.5. Animal Experiments

Male Sprague-Dawley (SD) rats (body weight 220 ± 50 g) were purchased and adapted under 65% RH, 23–27°C. The animal handling procedures were approved by the Institutional ethical committee and conformed to the principles of the International Guide for the Care and Use of Laboratory Animals. Eighteen rats were randomly divided into low (0.24 g/kg), medium (0.82 g/kg), high (2.45 g/kg) dose groups. The SD rats were fasted for 12 h before experiment, had free access to water even during the experiment. The blood (0.25 mL) was collected from the orbital venous plexus at 0.08, 0.25, 0.50, 1, 1.5, 2, 3, 4, 6, 8, 12 and 24 h after oral administration of *R. officinalis* extract. The plasma was immediately acquired by centrifugation at 4 °C and stored frozen at −20 °C until analysis.

### 3.6. Plasma Sample Preparation

Before the experiment, the plasma samples stored in −20 °C were thawed naturally at room temperature. Using the optimized method, 50 μL of I.S. (2120 ng/mL), 100 μL of methanol and 100 μL of 1 mol/L HCl were added to 100 μL of plasma sample followed with vortex-mixed for 30 s. The mixed sample was extracted with 3 mL of ethyl acetate by being vortex-mixed for 1 min. The upper organic layer was removed and evaporated to dryness under a stream of nitrogen at 40 °C after centrifuging at 3800 rpm for 5 min. The residue was reconstituted with 100 μL of acetonitrile-water (40:60, *v/v*), and then vortex-mixed for 2 min and filtered by a 0.22 μm membrane. 10 μL of the subsequent filtrate were injected into the UHPLC-ESI-MS/MS system [20].

### 3.7. Method Validation

The current UHPLC-ESI-MS/MS assay was validated for selectivity, linearity, precision, accuracy, extraction recovery, matrix effect and stability in accordance to the FDA guidelines, and was performed for the assay in the plasma of rats, http://www.fda.gov/downloads/Drugs/ GuidanceCompliance RegulatoryInformation/Guidances/UCM368107.pdf [21].

#### 3.7.1. Selectivity

Selectivity is the ability of an analytical method to differentiate and quantify the analytes in the presence of other components in the sample. In this paper, the selectivity was ascertained by comparatively analyzing blank plasma samples from six individual rats, corresponding blank plasma spiked with the three analytes and I.S. and the plasma samples from the rats after oral administration of the *R. officinalis* extract.

#### 3.7.2. Linearity of Calibration Curves and Lower Limits of Quantification (LLOQ)

The calibration curves were constructed by plotting the peak area ratios of each analyte to I.S. versus plasma concentrations on the basis of weighted linear least-squares regression model (1/*x^2^*). At the lowest analytical concentration on the calibration curve (LLOQ), the measured precision expressed as relative standard deviation (RSD) was required to be within 20%, and the accuracy expressed as relative error (RE) was required to be within ± 20% with an S/N of at least 10.

#### 3.7.3. Accuracy and Precision

The intra- and inter-day precision and accuracy were determined by testing the LLOQ sample and QC samples at three concentration levels (LQC, MQC and HQC) of the three analytes in six replicates each day for three consecutive days, respectively. The precision was determined and expressed as RSD and the accuracy as the RE. The acceptable criteria for the intra-day and inter-day precision and accuracy were within 15%.

#### 3.7.4. Extraction Recovery and Matrix Effect

The extraction efficiency of the three analytes was determined by analyzing six replicates of plasma samples at LQC, MQC and HQC levels. The recovery was evaluated by comparing the peak areas of the three analytes from the QC samples with those obtained from blank plasma samples with the three analytes spiked into the post-extraction supernatant. The matrix effect was evaluated by comparing the absolute peak areas of blank matrix samples spiked after extraction to the absolute peak areas of the unextracted samples. The extraction recovery and the matrix effect were similarly evaluated for I.S. at one concentration.

#### 3.7.5. Stability

The stability including cycles of freeze-thaw stability (three freeze at −20 °C and thaw cycles), room temperature stability (storage for 4 h at ambient temperature), long-term stability (storage for 2 weeks at −20 °C), post-preparation stability (storage for 12 h after sample preparation at 4 °C) were tested at LQC, MQC, HQC levels with six replicates at each level. The results were compared with those for freshly prepared QC samples and the percentage concentration deviation was calculated. The accuracy should be within ± 15% in freshly prepared samples. All the testing QC samples of stability were determined by using the calibration curve of freshly prepared standard samples.

### 3.8. Application to Pharmacokinetic Study

The maximum plasma concentration (*C*_max_) and the time of maximum plasma concentration (*t*_max_) were observed directly from the measured data. The elimination rate constant (*Ke*) was calculated by linear regression of the terminal points in a semi-log plot of the plasma concentration against time. The elimination half-life (*t*_1/2_) was calculated using the formula *t*_1/2_ = 0.693/*Ke*. The area under plasma concentration-time curve (AUC_0→t_) to the last measurable plasma concentration (*C*_t_) was estimated by using the linear trapezoidal rule. The area under the plasma concentration-time curve to time infinity (AUC_0→∞_) was calculated as AUC_0→∞_ = AUC_0→t_ + C_t_/*K_e_*. Pharmacokinetic parameters of the analytes were calculated with DAS 2.0 (Shanghai, China) by non-compartmental analysis.

## 4. Conclusions

This described UHPLC-MS/MS method was sensitive, accurate and fast, which met all requirements for bioanalysis. This is the first report of pharmacokinetic studies of carnosic acid, carnosol, rosmanol together in vivo following the oral administration of *R. officinalis* extract. The results would be helpful to provide some references to clinical application of this herb.

## Figures and Tables

**Table 1:** MRM transition in negative ion mode, and collision energy, quantifier and qualifier ions for the determination of the analytes and I.S.

No.	Compound	Transition	Fragmentor (V)	Collision Energy (eV)	Quantifier Ions	Qualifier Ions
1	Carnosic acid	331.2→287.2	120	20	287.2	244.1
2	Carnosol	329.1→285.1	120	11	285.1	200.9
3	Rosmanol	345.1→283.0	184	24	283.0	227.0
4	Butylparaben	193.0→92.0	110	20	92.0	136.0

**Table 2:** The regression equations, linear ranges and LLOQs for the determination of the analytes in rat plasma.

Compounds	Regression Equation	*R*^2^	Linear Range (ng/mL)	LLOQ (ng/mL)
Carnosic acid	*Y* = 0.069522*X* + 0.003893	0.9984	10.75~32,250	10.75
Carnosol	*Y* = 18.04392*X* + 0.048801	0.9982	1.453~4360	1.453
Rosmanol	*Y* = 3.279425*X* + 0.001014	0.9962	1.700~5100	1.700

**Table 3:** Intra-day and inter-day precisions and accuracies for the determination of the three diterpenes from the assay samples.

Compounds	Spiked Conc (ng/mL)	Measured Conc (ng/mL)	Accuracy (%)	Intra-Day Precision (%)	Inter-Day Precision (%)
Carnosic acid	10.75	10.60 ± 1.15	−1.39	11.30	6.44
	21.50	22.58 ± 2.09	5.02	8.72	12.47
	1075	1124 ± 65.39	4.53	5.88	5.37
	25800	27905 ± 2440	8.16	8.52	10.29
Carnosol	1.453	1.58 ± 0.20	8.39	12.60	14.37
	2.910	3.11 ± 0.26	7.09	7.48	12.70
	145.3	151 ± 18.74	3.91	12.28	13.35
	3488	3490 ± 70.26	0.08	2.14	0.35
Rosmanol	1.700	1.85 ± 0.24	8.88	11.89	14.29
	3.400	3.55 ± 0.21	4.36	4.77	11.29
	170.0	159.7 ± 12.48	−6.06	7.56	9.53
	4080	4270 ± 197.5	4.65	3.85	8.42

**Table 4:** The stability of the three diterpenes in rat plasma under different storage conditions (*n* = 6).

Compounds	Spiked Conc. (ng/mL)	Stability (% RE)			
		Freeze-Thaw	Short-Term	Long-Term	Post-Preparative
Carnosic acid	21.50	14.04	10.21	13.78	13.89
	1075	14.82	14.80	14.30	14.10
	25,800	14.40	14.84	14.84	14.83
Carnosol	2.910	5.39	8.07	3.10	2.32
	145.3	8.40	−8.67	14.49	0.66
	3488	0.30	−0.79	−0.48	2.09
Rosmanol	3.400	4.65	5.01	7.17	−0.30
	170.0	−7.12	−8.16	−8.56	−7.65
	4080	−0.07	0.65	9.17	4.60

**Table 5:** The absolute recovery and matrix effect of three diterpenes and I.S. in rat plasma (*n* = 6).

Compounds	Spiked Conc. (ng/mL)	Recovery (%)	RSD (%)	Matrix Effect (%)	RSD (%)
Carnosic acid	10.75	84.10	1.96	103.5	9.27
	1075	88.47	2.74	105.3	5.65
	25800	92.60	1.67	101.4	2.52
Carnosol	2.910	84.61	2.85	104.1	1.74
	145.3	89.29	2.23	105.3	2.39
	3488	93.41	3.44	103.6	3.09
Rosmanol	3.400	88.43	4.30	104.8	7.39
	170.0	89.30	2.25	105.3	2.40
	4080	95.14	1.87	102.1	1.49
IS	2120	82.64	5.58	97.34	10.01

**Table 6:** Pharmacokinetic parameters of three diterpenes after oral administration of *R. officinalis* extract at doses of 0.24 g/kg (*n* = 6), 0.82 g/kg (*n* = 6), and 2.45 g/kg (*n* = 6) to rats.

Analytes	Dose of Rosemary (g/kg)	*C*_max_ (ng/mL)	*t*_max_ (h)	*t*_1/2_ (h)	AUC_0→t_ (ng h/mL)	AUC_0→∞_ (ng h/mL)
Carnosic acid	0.24	1212 ± 344.23	0.30 ± 0.11	8.02 ± 2.08	9142 ± 1504	14,922 ± 10,211
	0.82	3955 ± 515.85	0.30 ± 0.11	12.81 ± 5.14	28,490 ± 2928	40,037 ± 32,222
	2.45	27,504 ± 1881	0.50 ± 0.31	12.84 ± 1.23	145,707 ± 12774	181,076 ± 19,700
Carnosol	0.24	216.56 ± 53.09	0.40 ± 0.13	12.29 ± 2.36	1466 ± 190.18	1819 ± 327.06
	0.82	413.34 ± 42.59	0.27 ± 0.15	13.86 ± 6.95	3187 ± 316.05	7358 ± 2738
	2.45	1480 ± 266.45	0.70 ± 0.45	12.83 ± 3.15	13,993 ± 3895	17,428 ± 4042
Rosmanol	0.24	77.20 ± 10.54	0.25 ± 0.00	8.88 ± 3.52	460.48 ± 54.92	552.10 ± 130.19
	0.82	187.90 ± 75.87	0.20 ± 0.18	9.75 ± 2.44	678.44 ± 56.94	825.96 ± 137.51
	2.45	633.41 ± 118.52	0.55 ± 0.27	15.36 ± 3.54	5369 ± 927.05	7985 ± 1648
